# Relationship between the Peroxidation of Leukocytes Index Ratio and the Improvement of Postprandial Metabolic Stress by a Functional Food

**DOI:** 10.1155/2016/5630985

**Published:** 2015-12-28

**Authors:** Ilaria Peluso, Husseen Manafikhi, Raffaella Reggi, Yaroslava Longhitano, Christian Zanza, Maura Palmery

**Affiliations:** ^1^Center of Nutrition, Council for Agricultural Research and Economics (CREA-NUT), Via Ardeatina 546, 00178 Rome, Italy; ^2^Department of Physiology and Pharmacology “V. Erspamer”, “Sapienza” University of Rome, Piazzale Aldo Moro 5, 00185 Rome, Italy

## Abstract

For the first time, we investigated the relationship between postprandial dysmetabolism and the Peroxidation of Leukocytes Index Ratio (PLIR), a test that measures the resistance of leukocytes to exogenous oxidative stress and their functional capacity of oxidative burst upon activation. Following a blind, placebo controlled, randomized, crossover design, ten healthy subjects ingested, in two different occasions, a high fat and high carbohydrates meal with Snello cookie (HFHCM-S) or with control cookies (HFHCM-C). Snello cookie, a functional food covered by dark chocolate and containing glucomannan, inulin, fructooligosaccharides, and* Bacillus coagulans* strain GanedenBC30, significantly improved postprandial metabolic stress (insulin, glucose, and triglycerides) and reduced the postprandial increase of uric acid. HFHCM-S improved PLIR of lymphocytes, but not of monocytes and granulocytes. Both meals increased granulocytes' count and reduced the lipoperoxidation induced by both exogenous free radicals and reactive oxygen species (ROS) produced by oxidative burst. Our results suggest that the healthy status of the subjects could be a limitation of this pilot study for PLIR evaluation on cells that produce ROS by oxidative burst. In conclusion, the relationship between PLIR and postprandial dysmetabolism requires further investigations.

## 1. Introduction

Postprandial dysmetabolism has been linked to atherosclerosis and inflammation [1]. Therefore, fatty meal consumption represents a model of acute inflammatory response and has been applied to study the effect of antioxidant-rich foods, beverages, or nutritional supplements, but results are scarce and controversial [2, 3].

Despite the fact that the consumption of high fat and high carbohydrates meals (HFHCM) has been associated with oxidative stress and with a decline in antioxidant defences in plasma, increases in plasma nonenzymatic antioxidant capacity have been reported following HFHCM [4, 5]. Furthermore, in healthy subjects, both increased [6] and reduced [7] reactive oxygen species (ROS) generation were observed in peripheral blood mononuclear cells (PBMC) during the postprandial period.

In this context, although oxidative stress is involved in metabolic syndrome, decreases in oxidative burst of neutrophils occurred in some conditions, such as hypercholesterolaemia [8] and non-insulin-dependent diabetes mellitus (NIDDM) [9].

Based on the potential protective effects against the onset of metabolic syndrome [10], functional foods containing probiotics, prebiotics, and/or polyphenols were placed on the market. Improvement of metabolic profile, oxidative stress, and inflammation has been reported also for glucomannan [11, 12].

de Luis et al. [13] pointed out that one of the problems of dietetic therapy is the lack of patient adherence and suggested that one possibility to overcome this problem is to include functional cookies in the diet. In particular, significant decreases of cholesterol and C reactive protein were observed in obese patients after the consumption of an alpha linolenic acid, fructooligosaccharides (FOS), and inulin-enriched cookie [13]. Improvements of glycemic control and lipid profile have been reported after consumption of glucomannan-enriched biscuits in subjects with impaired glucose tolerance, reduced high density lipoprotein (HDL) cholesterol, elevated serum triglycerides, and moderate hypertension [14].

In this pilot study, we aimed to investigate the relationship between the improvement of postprandial dysmetabolism by a functional cookie, covered by dark chocolate and containing glucomannan, inulin, FOS, and* Bacillus coagulans* strain GanedenBC30 [15], and the Peroxidation of Leukocytes Index Ratio (PLIR), a test that measures the resistance of leukocytes to exogenous oxidative stress and their functional capacity of oxidative burst upon activation [16].

## 2. Methods

### 2.1. Subjects' Selection

We recruited 10 healthy subjects who volunteered in response to advertisements.

Selection of subjects was made according to the following criteria: being healthy, being aged between 25 and 50 years, and taking no drugs, supplements, probiotics, or functional foods. Exclusion criteria include smoking habits and adherence to special diets (vegetarian, vegan).

Systolic Blood Pressure (SBP), Diastolic Blood Pressure (DBP), and heart rate (HR) were measured. MEDScore [17, 18] was calculated by the MedDietScore Software [19], and physical activity was calculated according to the “Guidelines for Data Processing and Analysis of the International Physical Activity Questionnaire” (IPAQ) [20, 21].

### 2.2. Study Design and Meals' Composition

For 2 days prior to each feeding study, the subjects followed a low antioxidant and low purine diet (washout), by avoiding fresh fruit and vegetables with high antioxidants' content and their products (juices or soups), tea, cocoa, nuts, coffee and wine, meat, and fish. Subjects were asked to refrain from exercise 2 days before the study. Compliance with dietary instructions was evaluated through dietary records and all subjects ingested pasta (160 ± 40 g/day), white bread (160 ± 50 g/day), croissant or cookies (50 ± 10 g/day), eggs (1/2 days), cheese (70 ± 20 g/day), fresh cheese (150 ± 50 g/day), milk (200 ± 50 mL/d), only a fruit/day (apple or pear), and less than 60 g/d of vegetables (zucchini, endive, or fennel).

Following a blind, placebo controlled, randomized, crossover design, subjects were allocated into Group A (*n* = 5) (HFHCM + control cookies) (HFHCM-C) or Group B (*n* = 5) (HFHCM + Snello cookie) (HFHCM-S). Panna cotta with caramel and control cookies were purchased from the supermarket. Snello cookie, a functional food commercially available in Italy, covered by dark chocolate and containing glucomannan (2.4 g/48 g), inulin (2.3 g/48 g), FOS (0.2 g/48 g), and* Bacillus coagulans* strain GanedenBC30 (3 × 10^6^ UFC/g), was provided by Nutripharma S.r.l.

After 12 days, subjects followed again 2 days of washout and 14 days after the first test the groups were crossed over to the alternative cookies. The macronutrient composition of the two meals, given as breakfast with 500 mL of water, is depicted in Table 1.

In patients with coronary artery disease (CAD) [22] or type 2 diabetes (T2D) [23], 2-hour breakfast tests [22, 23] revealed the improving effects of lipid lowering (fibrate) [22] or oral hypoglycaemic (mitiglinide) [23] drugs on the phorbol ester-activated leukocyte ROS production [22], plasma malondialdehyde (MDA), oxidized low density lipoproteins (oxLDL), plasma total radical-trapping antioxidant parameter (TRAP), and inflammatory cytokines [23]. Notwithstanding the above, the Ethics Committee approved a 3-hour test meal considering that ROS generation by polymorphonuclear (PMN) cells reached a peak 3 hours after meal in healthy subjects [6].

On the day of the study, after an overnight fast, venous blood samples were collected before (*T*0) and 30 minutes (*T*0.5), 2 hours (*T*2), and 3 hours (*T*3) after meal.

### 2.3. Clinical Markers

Blood was collected in Silicone-Coated tubes. The serum was stored at −80°C.

Serum levels of triglycerides (TG), glucose (GLU), and uric acid (UA) were quantified enzymatically using colorimetric kits (Sentinel CH. SpA, Italy). Plasma insulin was measured with an enzyme-linked immunosorbent assay (ELISA) kit (Li StarFish S.r.l., Italy).

### 2.4. PLIR Method

Blood was collected in EDTA tubes. After red blood cells' lysis and 4,4-difluoro-5-(4-phenyl-1,3-butadienyl)-4-bora-3a,4a-diaza-s-indacene-3-undecanoic acid (C11-BODIPY, Invitrogen, final concentration 1 *μ*M) staining, leukocytes were treated as previously described [24] with phorbol 12-myristate 13-acetate (PMA, Sigma, final concentration 1 *μ*g/mL), 2,2′-azobis(2-methylpropionamidine) dihydrochloride (AAPH, Sigma, final concentration 10 mM), 6-hydroxy-2,5,7,8-tetramethylchroman-2-carboxylic acid (Trolox, Sigma, final concentration 10 *μ*M), PMA 1 *μ*g/mL + Trolox 10 *μ*M, or AAPH 10 mM + Trolox 10 *μ*M. After 30 min at 37°C, cells were stored in ice, to stop reactions, and rapidly analyzed on an Accuri C6 BD cytometer.

**Figure 1:**
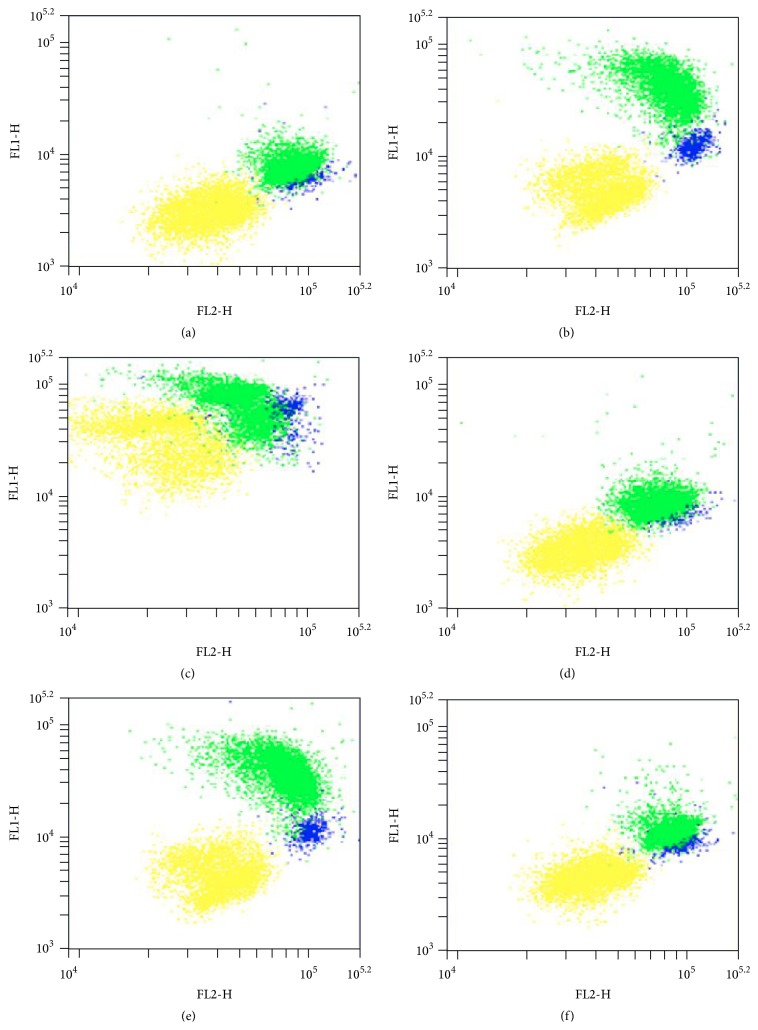
Typical dot plots of C11-BODIPY red (FL2) and green (FL1, oxidized) fluorescence of lymphocytes (yellow), monocytes (blue), and granulocytes (green) in unstimulated (UNST) samples (a) and after treatment with phorbol 12-myristate 13-acetate (PMA, (b)), 2,2′-azobis(2-methylpropionamidine) dihydrochloride (AAPH, (c)), 6-hydroxy-2,5,7,8-tetramethylchroman-2-carboxylic acid (Trolox, (d)), PMA + Trolox (e), or AAPH + Trolox (f).

C11-BODIPY, used in the PLIR method, modifies its fluorescence from red (FL2) to green (FL1) as a result of oxidation [25]. Treatment with AAPH or PMA changed the C11-BODIPY fluorescence in a different manner compared to unstimulated cells (Figure 1(a)), showing that oxidative burst induced ROS production only in activated cells (Figure 1(b)), while all cells were sensitive to exogenous (AAPH) ROS injury (Figure 1(c)). Trolox did not affect neither baseline levels of fluorescence (Figure 1(d)) nor the PMA-induced change in fluorescence of monocytes and granulocytes (Figure 1(e)) but decreased the AAPH-induced change in fluorescence of lymphocytes, monocytes, and granulocytes (Figure 1(f)).

Both FL1 and FL2 are higher in monocytes and granulocytes compared to lymphocytes in unstimulated samples (Figure 1(a)). Therefore, in order to normalize for cell incorporation of the probe into membrane, data acquired on the cytometer were exported in FCS format and analyzed by FCS Express software (De Novo Software) to calculate the ratio of oxidation of the probe C11-BODIPY (FL1/FL2). This ratio, being independent of the concentration of the probe, has been used to calculate PLIR, applying the previously described [16, 24] formula:

(1) $$\begin{array}{c} \text{PLIR}=\frac{\left(\text{RATIO AAPH}\times \text{RATIO PMA Trolox}\right)}{\left(\text{RATIO AAPH Trolox}\times \text{RATIO PMA}\right)}. \end{array}$$

PLIR is a functional index that measures the ratio between the resistance to exogenous (Trolox *μ*M equivalents AAPH) and resistance to endogenous (Trolox *μ*M equivalents PMA) ROS injury [16]. Although PLIR is independent of the baseline levels of oxidation, this functional index is sensitive to the difference between leukocytes isolated from fresh and stored blood [24].

Also Side Scatter (SS) was recorded and leukocytes' count was measured as previously described [26].

### 2.5. Statistics

Two-Way Repeated Measures Analysis of Variance (Two-Factor Repetition ANOVA), with cookies and time as within-subject factors, was performed. Student-Newman-Keuls post hoc analysis (All Pairwise Multiple Comparison Procedure) was used to isolate differences between groups.

All statistical evaluations were performed using the SigmaStat and Sigmaplot software (Jandel Scientific, Inc.).

## 3. Results

### 3.1. Characteristics of Subjects

Based on the exclusion criteria, ten subjects (6 men and 4 women), with a mean age of 36.0 ± 2.9 years and a mean body mass index of 23.3 ± 1.4, were recruited. Volunteers had a mean homeostasis model assessment of insulin resistance (HOMA-IR) of 1.6 ± 0.3 and were normotensive (SBP: 122.9 ± 3.1 mmHg, DBP: 75.9 ± 2.1 mmHg, and HR: 73 ± 2.5 beats/min).

Subjects had a MEDScore of 35.0/55 ± 1.9 (63.6% adherence's level to Mediterranean diet) [19] and a moderate physical activity (1089 ± 180 MET-minutes/week) [21].

### 3.2. Clinical Markers

Statistical analysis revealed a normal distribution for all markers (Normality Test Shapiro-Wilk passed: GLU: *p* > 0.8; INS: *p* > 0.5; TG: *p* > 0.8; UA: *p* > 0.9).

**Figure 2:**
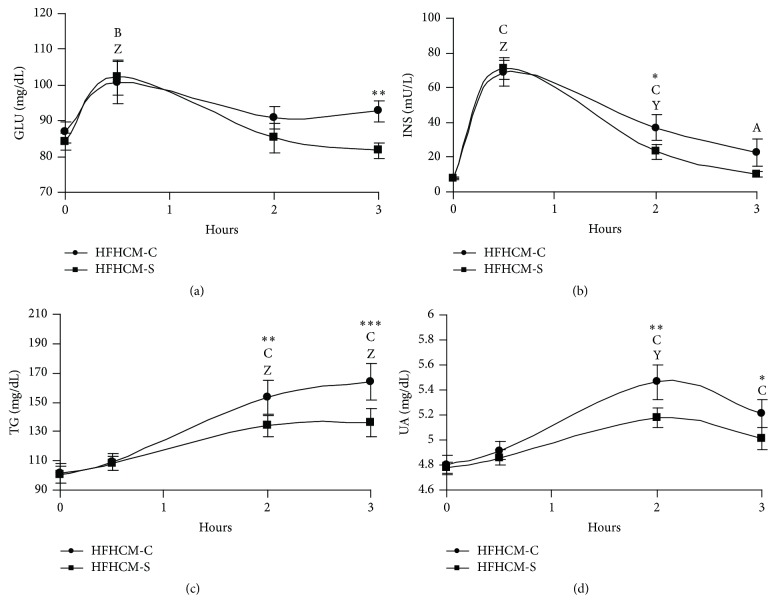
Line plots showing the serum levels as means ± standard errors (*n* = 10) in plasma glucose (GLU, (a)), insulin (INS, (b)), triglycerides (TG, (c)), and uric acid (UA, (d)), following high fat and carbohydrates meal ingestion with control (HFHCM-C) or Snello (HFHCM-S) cookies. Two-Way Repeated Measures ANOVA followed by Student-Newman-Keuls post hoc analysis. A: *p* < 0.05; B: *p* < 0.01; C: *p* < 0.001, single time point versus before meal intake within HFHCM-C; Y: *p* < 0.01; Z: *p* < 0.001, single time point versus before meal intake within HFHCM-S; ^*∗*^ *p* < 0.05; ^*∗∗*^ *p* < 0.01; ^*∗∗∗*^ *p* < 0.001, HFHCM-S versus HFHCM-C within time.

The glucose and insulin time courses reflected the postprandial load for healthy people. Both glucose and insulin levels peaked 30 min after meal ingestion (Figures 2(a) and 2(b)).

Although glucose and insulin levels began to decrease within one hour with both HFHCM-C and HFHCM-S, they returned to baseline values only with the latter (Figures 2(a) and 2(b)). In particular, glucose and insulin values were significantly higher after HFHCM-C compared to HFHCM-S at 3 hours (HFHCM-S versus HFHCM-C: *p* < 0.01; Figure 1(a)) and 2 hours (HFHCM-S versus HFHCM-C: *p* < 0.05; Figure 2(b)), respectively. Furthermore, insulin remained significantly above preingestion values for 3 hours only after HFHCM-C (*p* < 0.05 versus baseline; Figure 2(b)).

With respect to lipid metabolism, both HFHCM-C and HFHCM-S induced lipaemia at 2 and 3 hours; however, TG increase was significantly lower after HFHCM-S compared to HFHCM-C (HFHCM-S versus HFHCM-C: *p* < 0.01 within 2 hours, *p* < 0.001 within 3 hours; Figure 2(c)). Besides, HFHCM ingestion caused a significant increase in the endogenous antioxidant UA at 2 hours, but the latter was lower with HFHCM-S compared to HFHCM-C (within 2 hours: *p* < 0.01, within 3 hours: *p* < 0.05; Figure 2(d)). Furthermore, only HFHCM-C increased UA at 3 hours (*p* < 0.001 versus baseline; Figure 2(d)).

### 3.3. PLIR, Count, and Scatter of Leukocytes

Statistical analysis revealed a normal distribution for L, M, and G populations (Normality Test Shapiro-Wilk passed: L: *p* > 0.3; M: *p* > 0.2; G: *p* > 0.3).

**Figure 3:**
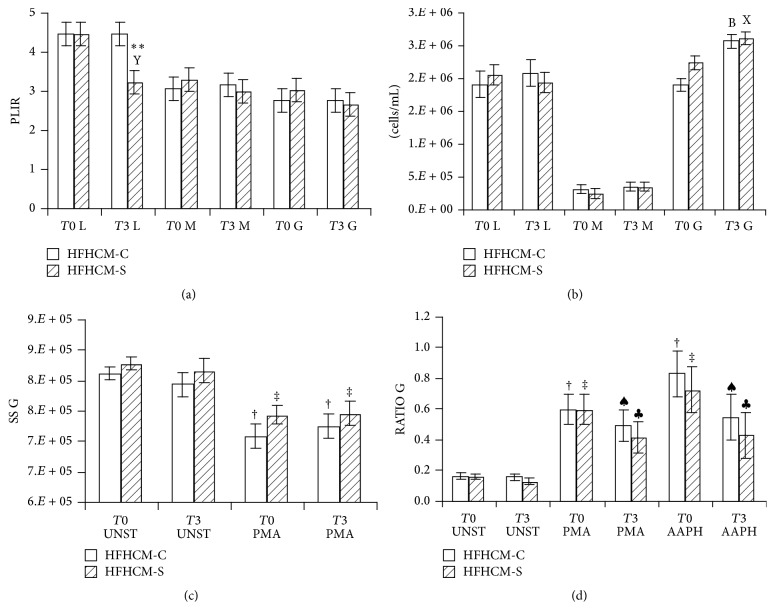
Vertical bars showing the values as means ± standard errors (*n* = 10) of PLIR (a), leukocytes count (b), Side Scatter of granulocytes (c), and RATIO of fluorescence (FL1/FL2) of granulocytes (d), following high fat and carbohydrates meal ingestion with control (HFHCM-C) or Snello (HFHCM-S) cookies. L: lymphocytes; M: monocytes; G: granulocytes. Two-way Repeated Measures ANOVA followed by Student-Newman-Keuls post hoc analysis. B: *p* < 0.01, 3 hours (*T*3) versus before (*T*0) meal intake within HFHCM-C; X: *p* < 0.05; Y: *p* < 0.01, 3 hours (*T*3) versus before (*T*0) meal intake within HFHCM-S; ^*∗∗*^ *p* < 0.01, HFHCM-S versus HFHCM-C within time; ^♠^ *p* < 0.01; ^†^ *p* < 0.001, treatment (PMA: phorbol 12-myristate 13-acetate; AAPH: 2,2′-azobis(2-methylpropionamidine) dihydrochloride) versus unstimulated (UNST) samples within HFHCM-C; ^*♣*^ *p* < 0.01; ^‡^ *p* < 0.001, treatment versus unstimulated (UNST) samples within HFHCM-S.

HFHCM-S, but not HFHCM-C, significantly decreased PLIR of lymphocytes at 3 hours (*p* < 0.01 versus baseline; HFHCM-S versus HFHCM-C: *p* < 0.01 within 3 hours; Figure 3(a)), whereas a nonsignificant decrease was observed for PLIR of monocytes and granulocytes after HFHCM-S (Figure 3(a)). In particular, considering the major components of PLIR affected by treatment, compared to baseline, the AAPH-induced (exogenous) oxidation of lymphocytes appeared significantly lower after HFHCM-S (difference of means RATIO AAPH versus RATIO UNST: at baseline 0.68, *p* < 0.001, at 3 hours 0.29, *p* < 0.01).

We analyzed also leukocytes' count and scatter after meal. The mean count and scatter from lymphocytes and monocytes remained unchanged after both meals. On the other hand, there was a postprandial increase in granulocytes' count with both meals (HFHCM-C versus baseline: *p* < 0.01; HFHCM-S versus baseline: *p* < 0.05; Figure 3(b)). However, this increase, after both meals, was accompanied neither by a reduction of SS in unstimulated samples nor by a different decrease in SS after PMA-activation (Figure 3(c)).

On the contrary, the increase in the RATIO of fluorescence of granulocytes after both PMA and AAPH treatment versus unstimulated samples appeared lower 3 hours after both meals (PMA or AAPH versus UNST: HFHCM-C at baseline: *p* < 0.001; HFHCM-C at 3 hours: *p* < 0.01; HFHCM-S at baseline: *p* < 0.001; HFHCM-S at 3 hours: *p* < 0.01; Figure 3(d)).

Similarly, nonsignificant effects were observed on monocytes with both meals.

## 4. Discussion

### 4.1. Postprandial Dysmetabolism

The functional food Snello cookie significantly improved postprandial metabolic stress. In particular, Snello cookie reduced the postprandial TG rise. Furthermore, glucose and insulin levels returned to baseline values at 3 hours after HFHCM-S, but not after HFHCM-C.

The effect on postprandial insulin could be due to the content of glucomannan in Snello cookie. In fact, McCarty [27] suggested that glucomannan reduces the postprandial insulin surge. Besides, results of a meta-analysis of randomized controlled trials pointed out that glucomannan significantly lowered TG and GLU [11].

On the contrary, a systematic review reported that studies evaluating the effects of inulin and FOS on glucose concentration in humans gave contrasting results [28]. On the other hand, inulin-enriched pasta [29] and breakfast cereal containing inulin [30] decreased TG. Inulin markedly increased bifidobacteria count and faecal concentration of lactate [30]. Lactic acid is involved in the immunomodulating effect of lactobacilli [31]. Although the prebiotic effects of inulin-type prebiotics, including FOS and inulin, occur after long term consumption [32], it has been reported that acute inulin ingestion increased postprandial serum short-chain fatty acids and reduced free fatty acids [33, 34].

On the other hand, also the chocolate contained in Snello cookie could improve postprandial dysmetabolism. Although acute cocoa supplementation showed no clear overall benefit on postprandial GLU, INS, and TG [35, 36], it has been reported that an oral supplement of (−)-epicatechin (the major flavanol contained in chocolate) significantly lowered GLU and TG 2 hours after meal [37].

Therefore, the overall improvement of postprandial dysmetabolism induced by Snello cookie could be due to the synergistic effect of its constituents.

### 4.2. Postprandial Leukocytes' Recruitment and Activation

We observed the previously described [38–41] postprandial increase of granulocytes' count at 3 hours. In this context, the leukocytes' excursion, after meal, was significantly reduced with acarbose in patients with T2D and subclinical inflammation (leucocytes > or = 6.2 gigaparticles/L) [42]. Rosiglitazone reduced the incremental area under the curves for leukocytes, due to a specific reduction of neutrophils (−39%, *p* < 0.05), in patients with T2D [43]. On the contrary, rosuvastatin did not affect baseline leukocytes' count or the postprandial neutrophils' increase in CAD patients [44]. Furthermore, results of statin withdrawal demonstrated that the expression of leukocytes' markers of activation is not affected by the use of statins [45]. However, the studies that investigated the effect of glucose on leukocytes' markers of activation have shown conflicting results in T2D [45–48]. On the other hand, postprandial studies reported that leukocytes are activated by lipids [38, 49, 50]. The expression of leukocytes' markers of activation (i.e., CD11b, CD11c) increased on monocytes or neutrophils after a high fat meal [50, 51]. The extent of upregulation of the expression of leukocytes' markers of activation correlated with TG and was accompanied by an altered scatter profile [50, 51]. In particular, CD11b expression on neutrophils was negatively correlated with the mean SS of neutrophils, reflecting granularity [50].

In our study, the increase in granulocytes' count was accompanied neither by a reduction of SS in unstimulated samples nor by a different decrease in SS after PMA-activation. In this context, the fact that, even if white blood cells' count increased and intracellular myeloperoxidase decreased within 2–4 hours after meal, waist-to-hip ratio influenced the degranulation of PMN must be taken into account [52]. Besides, recent results showed that only obese subjects had higher postprandial endotoxemia, the mechanism of postprandial leukocytes' activation, despite the lipaemia increased in both normal-weight and obese men after meal [53]. Furthermore, postprandial leukocytes' activation was highest in patients with T2D and hyperlipidaemia [45]. Therefore, our pilot study has the major limitation that subjects were healthy, of normal weight, with a moderate physical activity and none of them presented risk factors for CVD.

### 4.3. PLIR and UA in the Postprandial Phase

Snello cookie improved PLIR of lymphocytes, but not of monocytes and granulocytes. To understand this result further considerations should be made.

Although PLIR is a functional index that is independent of baseline levels of oxidation, measuring the ratio between the resistance to exogenous and resistance to endogenous ROS injury [16], this ratio calculation could mask the effect of foods that inhibit both the exogenous ROS injury and the oxidative burst. In particular, the calculation of PLIR includes the PMA-induced oxidation. Lower increases in the RATIO of fluorescence of granulocytes after both PMA and AAPH treatment versus unstimulated samples were recorded 3 hours after both HFHCM-C and HFHCM-S compared to baseline. However, the unchanged mean SS (reflecting granularity) after meal suggests that the decrease in the RATIO of fluorescence is more likely due to the antioxidant effect of UA and cocoa flavanols after HFHCM-C and HFHCM-S, respectively, rather than an effect on oxidative burst. In agreement with this hypothesis, Sodré et al. [7] reported that the intracellular ROS in PBMC, assessed by flow cytometry as the ethidium (ETH) fluorescence, decreased 2 and 4 hours after meal not only in monocytes but also in lymphocytes, which do not produce ROS by oxidative burst. On the contrary, others [6] reported that the release of superoxide radical by PMN, as measured by chemiluminescence, was significantly lower when orange juice was added to the meal than when water or glucose was added to the meal. However, extracellular free radicals' measurements, such as the chemiluminescence assay, are deeply affected by cell count and viability. Therefore, the postprandial increase of granulocytes' count [38–41] could bias these methods.

On the other hand, we observed an increase in UA after HFHCM-C. This increase could be due to the healthy status of the subjects. In agreement with this, increases in TRAP and UA have been reported following HFHCM in healthy subjects [4], despite the lower TRAP values after meal in T2D patients [23].

The increase in UA was lower after HFHCM-S. The inhibition of UA increase could be due to the cocoa flavanols contained in Snello cookie. In agreement with this hypothesis, tea flavanols could have UA lowering effect [54, 55] and fruit-based juice drinks, providing exogenous antioxidants, prevented the endogenous antioxidant response to HFHCM, by inhibiting the production of UA [56].

In this context, UA levels could affect PLIR in two different ways: acting as antioxidant [57] on all leukocytes and inducing oxidative burst in ROS-producing cells [58]. The effect of UA depends on its concentration. AAPH-induced lipid peroxidation, in vitro, was strongly inhibited by UA at concentration ranging between 50 and 400 *μ*M (0.84–6.72 mg/dL) [57]. The increase in UA after HFHCM-C ingestion could justify why HFHCM-C did not change PLIR. In this context, it has been suggested that in healthy people the body responds to postprandial stress by inducing endogenous defenses [4]. In the presence of dietary antioxidants (i.e., the chocolate contained in Snello cookie), the resistance to AAPH-induced oxidation is increased in lymphocytes despite the reduced UA increase.

On the other hand, although the level at which UA concentration becomes abnormal is still disputed, ranging between 3.5 and 7.2 mg/dL in adult males and postmenopausal women and between 2.6 and 6.0 mg/dL in premenopausal women [59], a threshold value below the saturation concentrations (<6 mg/dL or <360 *μ*mol/L), in order to prevent monosodium urate (MSU) crystals formation, has been suggested [59, 60]. In fact, in response to MSU, the neutrophils recruited to sites of inflammation undergo oxidative burst [58]. In our study, after HFHCM-C, UA reached the concentration of 5.46 ± 0.13 mg/dL, a value below the threshold value of 6 mg/dL [59, 60]. From that, the healthy status of the subjects could be a limitation of this study for PLIR evaluation on cells that produce ROS (i.e., monocytes and granulocytes).

## 5. Conclusion

In conclusion, the functional food Snello cookie significantly improved postprandial metabolic stress and reduced the postprandial increase of UA.

After HFHCM-S, PLIR was improved on lymphocytes, but not on monocytes and granulocytes.

The healthy status of the subjects could be a limitation of this pilot study for PLIR evaluation on cells that produce ROS (i.e., monocytes and granulocytes). From that, further studies on subjects who are at risk of cardiovascular diseases are needed in order to investigate the relationship between postprandial dysmetabolism and PLIR.

## Figures and Tables

**Table 1:** Macronutrient composition of the two meals.

	Kcal	Lipids (saturated)	Proteins	Carbohydrates (sugars)
HFHCM-C (total)	**831.4**	**40.9 (29.6)**	**6.9**	**105.7 (63.3)**
Panna cotta with caramel (240 g)	647.4	34.3 (27.4)	4.3	77.5 (54.5)
Control cookies (40 g)	184	6.6 (2.2)	2.6	28.2 (8.8)

HFHCM-S (total)	**846.4**	**40.6 (31.2)**	**6.8**	**108.8 (65.0)**
Panna cotta with caramel (240 g)	647.4	34.3 (27.4)	4.3	77.5 (54.5)
Snello cookie (48 g)	199	6.3 (3.8)	2.5	31.3 (10.5)

C: control cookies; HFHCM: high fat and high carbohydrates meals; S: Snello cookie.
